# Identification of Environmental Factors Influencing the Population of Endangered *Epilobium hirsutum* L. in Korea

**DOI:** 10.3390/biology13121061

**Published:** 2024-12-18

**Authors:** Rae-Ha Jang, Sunryoung Kim, Minhan Kim

**Affiliations:** 1 Biodiversity Research and Cooperation Division, National Institute of Biological Resources, Incheon 22689, Republic of Korea; rhjang@korea.kr; 2 Habitat Conservation Team, National Institute of Ecology, Yeongyang 36531, Republic of Korea; sun02@nie.re.kr

**Keywords:** endangered wildlife, wetland species, boreal plant, environmental factor

## Abstract

*Epilobium hirsutum* has been identified as a Class II endangered species in Korea. Therefore, there is an urgent need to develop habitat conservation and improvement strategies for the protection of *E. hirsutum*. In this study, we aimed to evaluate the environmental characteristics of *E. hirsutum* habitats and identify key environmental factors influencing the population size. To this end, we conducted field surveys and performed a spatial data analysis to collect data on 38 environmental characteristics associated with the population size of *E. hirsutum*. We performed factor and path analyses to identify the environmental factors influencing the population size and the correlations between the population size and environmental factors. The common factors identified in both analyses were the average temperature in March, available P, soil pH, and soil humidity. Therefore, to manage *E. hirsutum* habitats or create alternative habitats, areas with a low air temperature and high soil moisture must be selected. It is necessary to remove tall plants and amend soils with alkaline fertilizers containing P. Additionally, it may be effective to implement a management strategy that induces vegetative propagation and seed germination by replacing agricultural waterways made of concrete with those made of rock and soil.

## Simple Summary

In this study, we investigated the environmental characteristics of all known habitats of *Epilobium hirsutum* in Korea. Habitat characteristics are influenced by various environmental factors such as water, soil, and temperature. In this investigation, we analyzed the environmental factors influencing the population size of this species, which is classified as endangered in Korea. Our findings indicated that atmospheric and soil conditions had the greatest impact on the population size of *E. hirsutum*. These results serve as a valuable reference for developing management strategies for *E. hirsutum* habitats and provide foundational data for future habitat restoration and improvement efforts.

## 1. Introduction

In situ conservation methods have shown promising results for biodiversity conser-vation [1]. Among these methods, the designation of protected areas is widely recognized as one of the most effective approaches for global-scale biodiversity and habitat conservation [2,3]. The establishment of protected areas has contributed to the successful conservation and restoration of various animal and plant species in many regions worldwide [4,5].

In 2012, the Ministry of Environment in Korea designated *Epilobium hirsutum*, a member of the Onagraceae family, as a protected Class II endangered species. *E. hirsutum* holds significant importance in the context of biodiversity and ecological research in South Korea. Notably, in 2017, *E. hirsutum* was identified as a potential hybrid parent of *Epilobium ulleungensis*, J. M. Chung (Onagraceae Juss), a newly described species presumed to have originated from hybridization with *E. pyrricholophum* or *E. amurense* subsp. *cephalostigma*, drawing considerable attention from the scientific community [6]. Furthermore, the population of *E. hirsutum* has shown a noticeable decline compared to its historical distribution, highlighting the necessity for conservation measures. Maintaining and restoring species diversity is recognized as a critical component of national efforts to safeguard natural heritage.

As a C3 plant, *E. hirsutum* exhibits tolerance for low temperatures, which allows it to germinate and grow under cold conditions; however, it struggles to thrive in high temperatures (>30 °C) [7]. Populations of *E. angustifolium* thrive across a wide range of environments, from sea level in central and northern regions to elevations of 4870 m in the Himalayas, with a notable prevalence in the open and semi-open habitats of the boreal forest [8]. *E. hirsutum* is also widely distributed in cold regions such as high latitudes and alpine zones [7]. Notably, *E. hirsutum* occupies a small ecological niche, with its habitat range and distribution being quite limited [9,10].

Bioclimatically, most of South Korea falls within the cool, temperate, deciduous broad-leaved forest zone, with subalpine regions accounting for only about 0.9% of the total land area [11]. Furthermore, the area of forest grassland suitable for *E. hirsutum* is less than 1% of the total area [12]. Therefore, *E. hirsutum* has limited available habitats in Korea, leading to a small population size in the wild, and it is considered particularly vulnerable to climate change. Therefore, clear guidelines are required for designating protected areas. However, this approach has sometimes led to conflicts with local communities over property rights and restrictions on activities within protected lands [13].

In this study, we aimed to identify the environmental characteristics of *E. hirsutum* habitats where population numbers are continuously declining, providing foundational data for the designation of protected areas. Additionally, we aimed to analyze the correlation between population numbers and environmental factors to devise strategies for the improvement and conservation of *E. hirsutum* habitats. Through this research, we seek to clarify and detail the key factors for the conservation of native habitats, with the goal of minimizing conflicts with local communities and enhancing conservation efficiency during the designation of protected areas and population protection efforts.

## 2. Materials and Methods

### 2.1. Study Area

**Figure 1:**
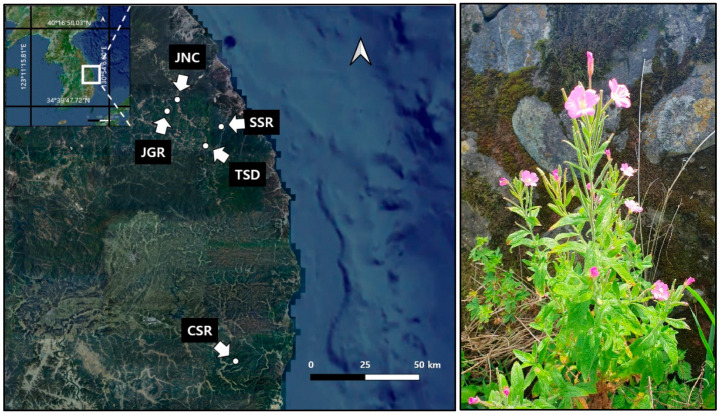
Map of *Epilobium hirsutum* study sites and a photo of the species. To prevent illegal harvesting of the legally protected species, detailed locations are not disclosed.

Based on the results of the Ministry of Environment’s “National Survey on Endangered Wildlife Distribution”, we identified the coordinates of 11 sites where *E. hirsutum* grows in Korea. To clearly identify individual plants, we conducted field surveys during the flowering season and confirmed the distribution at 6 of the 11 sites. Due to severe disturbance from development, the *E. hirsutum* habitat on Ulleung Island was substantially damaged and was, therefore, excluded from the study. Consequently, all *E. hirsutum* habitats located inland were selected as research sites. These sites were located at: (1) Sangsami-dong, Taebaek-si, and Gangwon Province (TSD), (2) Goyang-ri, Imgye-myeon, Jeongseon-gun, and Gangwon Province (JGR), (3) Naktcheol-ri, Imgye-myeon, Jeongseon-gun, and Gangwon Province (JNC), (4) Seohari, Singi-myeon, Samcheok-si, Gangwon Province (SSR), and (5) Sangui-ri, Juwangsan-myeon, Cheongsong-gun, and Gyeongsangbuk Province (CSR) (Figure 1).

### 2.2. Meteorological Data Collection and Spatial Mapping

To investigate the meteorological characteristics of the study sites, we used climate data from WorldClim (https://www.worldclim.org/, accessed on 15 September 2023) for the average temperatures and water vapor pressures from 1970 to 2000, which were most recently provided in January 2020. The maximum temperature, minimum temperature, and precipitation data were provided monthly, and the averages over the last 10 years (2011–2022) were used. To analyze the spatial data of each study site, ArcGIS Pro 3.1.0 was used.

### 2.3. Field Survey Data Collection

The field survey was conducted using a small quadrat of 1 m × 1 m, considering that the target species was a herbaceous plant. Three squares were installed at each site, with the target species in the center, so that they did not overlap. We recorded the number of individual plants within each quadrat. At SSR and CSR sites with a population of less than three individuals, additional quadrats were installed adjacent to quadrats centered around *E. hirsutum* under similar environmental conditions. The soil pH and humidity were measured using an acid hygrometer (DM-5, Takemura, Kyoto, Japan). Soil samples were collected at a depth of 10 cm to analyze the organic matter content, available P, exchangeable cations (K, Ca, Mg, and Na), electrical conductivity, and soil texture (sand, silt, and clay contents). Soil classification was based on the proportions of sand, silt, and clay according to the soil taxonomy system of the United States Department of Agriculture. Water quality analysis involved measuring the dissolved oxygen, pH, and electrical conductivity three times each using a portable multiparameter water quality meter (YSI-pro20, YSI, Yellow Springs, OH, USA).

### 2.4. Statistical Analysis

To identify the major factors influencing the abundance of *E. hirsutum*, factor analysis of the collected environmental variables was conducted using Statistica software (version 7, TIBCO Software Inc., Palo Alto, CA, USA). To prevent bias caused by differences in the scales of the variables, normalization of the item variables was performed using Statistica software (version 7, TIBCO Software Inc.) prior to conducting the factor analysis. Additionally, path analysis was conducted to investigate the correlations between the number of *E. hirsutum* individuals and environmental factors, as well as the relationships among environmental factors. This analysis was performed using JASP statistical software (version 0.18.0.0, Amsterdam University, Amsterdam, The Netherlands).

## 3. Results

### 3.1. Meteorological Data

**Figure 2:**
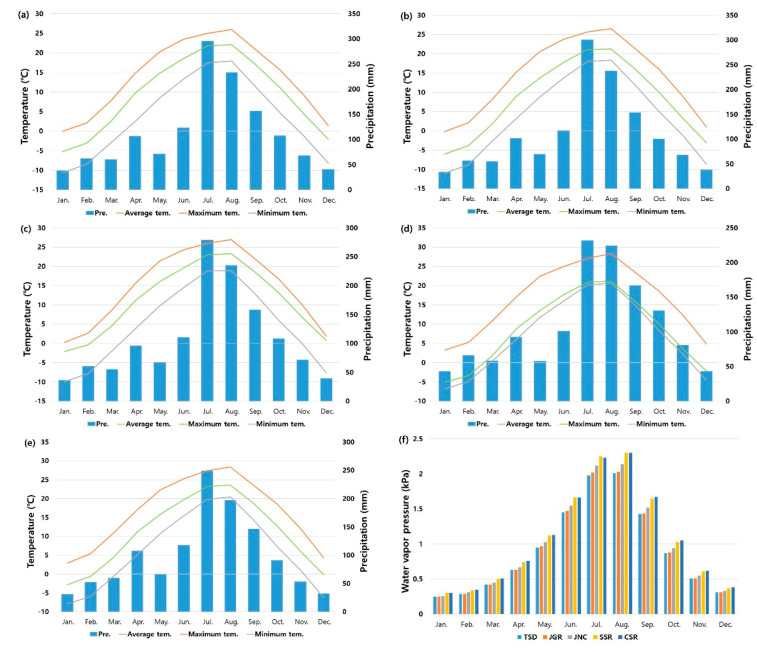
Annual climate diagrams for (**a**) TSD, (**b**) JGR, (**c**) JNC, (**d**) SSR, and (**e**) CSR and (**f**) monthly water vapor pressure by research site. The red line represents the highest temperature, the green line represents the average temperature, and the gray line represents the lowest temperature.

The annual average temperature across the five study sites was 9.7 °C, with a maximum of 15.2 °C and a minimum of 5.1 °C. Variations in average temperatures and the extremes of the coldest and hottest temperatures followed similar patterns across all regions. The annual maximum temperatures ranged from 25 °C to 30 °C, while minimum temperatures dropped below −10 °C in the TSD, JGR, and JNC regions and ranged from −5 °C to −10 °C in the SSR and CSR regions (Figure 2).

Reflecting the climatic characteristics of South Korea, approximately 50% of the annual precipitation occurred between July and September, with a total annual precipitation of 1305.6 mm. The highest monthly rainfall typically occurred in July, ranging from 250 mm to 300 mm in most regions. However, the SSR region recorded less than 250 mm in July, indicating relatively lower precipitation levels (Figure 2).

The annual average water vapor pressure was 1 kPa, with a total annual water vapor pressure of 12 kPa. In terms of the monthly variation, TSD and JGR consistently exhibited the lowest water vapor pressure values, while SSR and CSR showed the highest levels throughout the year, with JNC displaying intermediate values (Figure 2f).

### 3.2. Field Survey Results

The highest abundance of individuals was observed in the TSD region, while the other three regions exhibited fewer than five individuals. The height of the herbaceous layer was similar across all regions. The distance from woody plants was approximately 10 m in the TSD and SSR regions, while it was the shortest in the JGR region at 2 m.

The distance from water bodies was less than 20 cm in the TSD, JGR, and SSR regions, indicating their proximity to water sources, while the JNC and CSR regions were also located near water bodies, with distances of approximately 3 m. The width of the waterline was widest in the CSR region at 15.33 m, whereas it was less than 2 m in the other regions. Rock exposure was the highest in the SSR region, while it was below 10% in the remaining regions.

Slope steepness was greatest in the TSD and SSR regions, with the other regions exhibiting gentler slopes of less than 14°. Regarding soil depth, the TSD region recorded the deepest soil at 28.3 cm, followed by the JGR and JNC regions with depths exceeding 20 cm. In contrast, the SSR and CSR regions had relatively shallow soils, measuring around 10 cm (Table 1).

The soil of the five *E. hirsutum* study sites showed an average pH of 6.62, organic matter content of 3.7%, and available P content of 99.01 mg/kg. The concentrations of exchangeable cations in the soil were K 0.32 cmol^+^/kg, Ca 10.95 cmol^+^/kg, Mg 1.48 cmol^+^/kg, and Na 0.19 cmol^+^/kg, and the average cation exchange capacity was 10.73 cmol^+^/kg. The electrical conductivity was 117.5 µS/cm, and the soil was composed of 77.58% sand, 12.07% clay, and 10.35% silt (Table 2). In terms of water quality, the dissolved oxygen was 9.38 mg/L, pH was 6.85, electrical conductivity was 261.16 µS/cm, and water pressure was 717.65 mmHg (Table 3).

### 3.3. Statistical Analysis Results

#### 3.3.1. Selection of Key Environmental Variables

**Figure 3:**
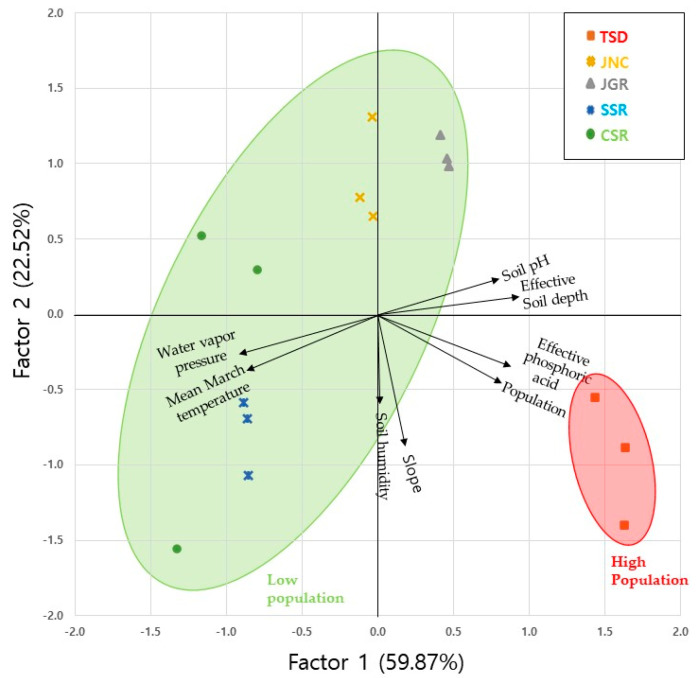
The results of factor analysis between the areas with high and low populations of *E. hirsutum*.

The results indicated that the abundance of *E. hirsutum* was influenced by the water vapor pressure, average March temperature, soil pH, effective soil depth, available phosphorus (P), soil moisture, and ground slope among the 38 variables analyzed (Figure 3, Table 4). The results of the factor analysis showed a clear distinction between high and low *E. hirsutum* abundance across the entire study area. As shown in Figure 3, sites with low *E. hirsutum* abundance were evenly distributed in Quadrants 1, 2, and 3, whereas sites with high abundance were located only in Quadrant 4.

#### 3.3.2. Path Analysis

**Figure 4:**
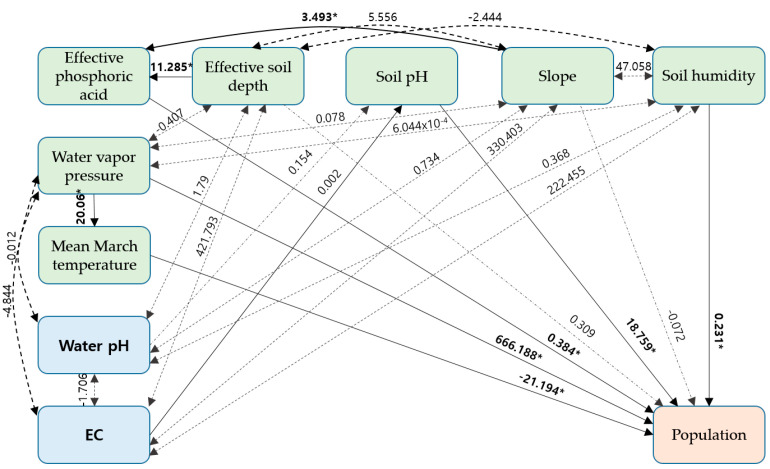
The result of pathway analysis between the areas with high and low populations of *E. hirsutum*. (Numbers above each arrow indicate path coefficients. *, *p* < 0.05).

Path analysis confirmed the correlation between *E. hirsutum* abundance and environmental factors (Figure 4). In particular, *E. hirsutum* abundance was positively correlated with the water vapor pressure (666.188 of β, *p* < 0.001), effective phosphoric acid (0.384 of β, *p* < 0.001), soil pH (18.759 of β, *p* < 0.001), and soil humidity (0.231 of β, *p* < 0.001). Notably, the relationship between abundance and the effective soil depth was not significant. The number of individuals was negatively correlated with the mean March temperature (−21.194 of β, *p* < 0.001), but the correlation with the slope was not significant. Effective phosphoric acid was positively related with the effective soil depth (11.285 of β, *p* < 0.001) and slope (3.493 of β, *p* < 0.001). The water vapor pressure (20.06 of β, *p* < 0.001) increased with the increasing mean March temperature. The soil pH was positively correlated with electrical conductivity (0.002 of β, *p* < 0.001). However, the relationship between the soil pH and stream pH was not significant.

## 4. Discussion

To identify the main environmental factors affecting the population of *E. hirsutum*, 38 factors, including vegetation, soil quality, and water quality, were examined at the five *E. hirsutum* habitat sites. The results of the field survey showed that all *E. hirsutum* sites were located in an unshaded environment with no tall, woody plants in close proximity and that the distance from shrubs was at least 2 m. Sunlight is an important environmental factor for *E. hirsutum*, which requires a photoperiod exceeding 14 h; a lack of sunlight leads to poor growth and low germination rates [7,15,16]. Habitat shading is a threat to certain herbaceous plants [17], and *E. hirsutum* has been reported to avoid habitat shading by nearby woody plants, instead preferring areas with abundant sunlight. 

Factor analysis confirmed that, among the 38 variables examined, the mean March temperature, soil pH, effective soil depth, available P, soil humidity, and slope were identified as the habitat factors affecting *E. hirsutum* abundance (Figure 3). A path analysis also showed that the abundance of *E. hirsutum* was strongly correlated (*p* < 0.001) with the mean March temperature (Figure 4). Notably, the mean March temperature of the study site was 7.7–11 °C, which aligns with the minimum germination temperature of *E. hirsutum* (8 °C) [7]. In Korea, which is located in a temperate forest biome [18], the ability to germinate at low temperatures may serve as an advantage when competing with other plant species for habitats.

The abundance of *E. hirsutum* was also positively correlated with the soil humidity and water vapor pressure. Field surveys revealed that *E. hirsutum* habitats were distributed linearly along streams, with areas of high soil humidity and partial inundation during the rainy season. Globally, *E. hirsutum* is restricted to wet swamps, coastal areas, stream banks, and ditches, and it grows better at higher soil moisture levels than at lower levels [7,8,9,10]. Plant growth at similar latitudes depends on the temperature and annual precipitation, and plants that grow in colder regions, such as *E. hirsutum*, produce approximately three times as much for the same amount of water as plants that live in warmer regions [19].

In addition, an increased water vapor pressure generally promotes transpiration, which requires more humidity. If the supply of moisture is insufficient, desiccation damage occurs, or the stomata close to prevent desiccation, reducing stomatal conductance and the transpiration rate [20]. As transpiration in plants occurs through the stomata, there is a close relationship between stomatal conductance and the transpiration rate [20]. A decrease in stomatal conductance leads to a decrease in the photosynthetic capacity owing to a decrease in the concentration of CO_2_ in the chloroplasts, which in turn leads to a decrease in the metabolism, resulting in stunting and death [21,22]. As stomatal conductance increases with an increase in the transpiration rate, *E. hirsutum* may suffer from burns due to excessive light energy if it does not have sufficient soil moisture [23,24]. Therefore, *E. hirsutum*, which has a narrow ecological range for moisture [10], requires both sufficient light intensity and high soil humidity to grow during the hot and humid summers in Korea.

The soil pH of the *E. hirsutum* habitat was found to be between 6.23 and 6.9, and a path analysis revealed that the abundance of *E. hirsutum* was positively correlated with the soil pH. These results were similar to previous findings that *E. hirsutum* seeds do not germinate in soils with a pH below 6.5 and prefer near-neutral soils with a pH between 6.8 and 7.5 [9,16]. The CSR site, with an identified soil pH of 6.23, supported more than 100 *E. hirsutum* individuals in 2014 and 2017, but only three individuals were found in 2022 and one in 2023. Although there may be many complex reasons for this dramatic decline, it is likely that the area has experienced soil acidification, making it unsuitable for *E. hirsutum*.

Except in JNC, the higher the soil organic matter content, the higher the population of *E. hirsutum*. The soil organic matter content is positively correlated with total N in the soil [25], and *E. hirsutum* grows in nutrient-rich soils [15]. Furthermore, increasing the soil organic matter content has a positive effect on *E. hirsutum* growth by accelerating its flowering and fruiting times [10].

The abundance of *E. hirsutum* was also positively correlated with the available P content of the soil. Along with N and K, P is one of the three main elements required for plant growth, and it is essential for energy transportation and root growth. However, the relationship between the P content and *E. hirsutum* growth has not yet been studied. In nature, the nutrient use efficiency of plants is highly variable, depending on limiting factors such as the availability of water and micronutrients [19]. Generally, P is poorly available in both terrestrial and aquatic environments, and its availability is strongly influenced by soil pH [26]. In acidic soils, P is strongly bound to clay and forms near-insoluble P compounds with Fe and Al, whereas in basic soils, P is bound to Ca to form another insoluble compound. Free H_3_PO_4_ is present at its highest concentrations at a pH between 6 and 7 [19]. For these reasons, a lack of P is a major limiting factor for plants growing in wetlands, and anthropogenic P inputs stimulate primary production [19]. Therefore, the distribution of *E. hirsutum* is likely influenced by the soil pH and available phosphorus levels. Notably, the field survey revealed that the TSD site hosted a significantly higher number of *E. hirsutum* individuals compared to other sites. This population difference may be attributed to the site’s unique location in an agricultural ditch situated between extensive Napa cabbage fields and near livestock barns, which likely contribute to the higher soil phosphorus content relative to other surveyed areas.

Meanwhile, the ground slope and effective soil depth were found to have weak correlations with *E. hirsutum* abundance. In the field survey, *E. hirsutum* was evenly distributed on ground slopes ranging from 0° to 28.3°. These results are similar to those of Kim [15] who found that *E. hirsutum* can be planted on slopes of up to 25° when creating alternative habitats. In the current study, the soil depth in the area where *E. hirsutum* was growing was shallower than 30 cm, and tall plants with deep roots could not settle in this location (Table 1). However, the results of the field survey confirmed that a steep ground slope hinders the retention of soil moisture. As the flow rate increases, soil shedding intensifies, making it difficult for *E. hirsutum* to grow in close proximity to streams with steep banks. 

Based on these results, to conserve and improve the habitat of *E. hirsutum*, it is necessary to manage the removal of tall plants to ensure sufficient sunlight [7,15,16,17]. Additionally, based on the results of the path analysis, it is important to maintain environments with high soil moisture, such as wetlands, to provide a continuous water supply to the habitat. Furthermore, in acidic soils, the application of alkaline fertilizers is essential to keep the soil pH between 6.5 and 7. Finally, P fertilizer application may also be considered as a management strategy in areas where competing species are under control.

## 5. Conclusions

This study was conducted to provide basic data for the conservation and improvement of the habitat of *E. hirsutum*, a Class II endangered species whose habitat in Korea has continuously decreased. Through field surveys and spatial analysis, 38 items of data on the *E. hirsutum* population and habitat environmental characteristics were collected, and the environmental factors affecting the population were derived and correlated through factor and path analyses. The main environmental factors affecting the abundance of *E. hirsutum* were the soil moisture, soil pH, available P, water vapor pressure, and the mean March temperature. The number of individuals was negatively correlated with the mean March temperature and positively correlated with other factors. Although the correlations were not significant, areas with deeper soil and gentler ground slopes tended to have larger populations.

Removing taller vegetation and managing the soil pH and nutrient levels can facilitate the germination and growth of *E. hirsutum* seeds. In Korea, *E. hirsutum* is predominantly found in irrigation ditches. However, most farm ditches are constructed with concrete and steep slopes, which limit the spread of plants into the surrounding areas and restrict suitable habitats. To preserve the habitat of *E. hirsutum*, replacing concrete-lined farm ditches with natural ditches composed of pebbles and soil is recommended. Such modifications would support the natural dispersal and vegetative propagation of the species.

Although reversing modern agricultural trends to protect a single species is impractical, various species in South Korea, such as *Pelophylax nigromaculatus, Dryophytes japonicus, Rana dybowskii*, and *Glandirana rugose*, Temminck and Schlegel, are experiencing habitat loss due to artificial agricultural waterways [27]. Additionally, endangered species like *Dryophytes suweonensis* and *Pelophylax chosenicus* face significant threats from these artificial structures [28,29]. Maintaining natural-style agricultural waterways in select regions could be a critical conservation strategy not only for *E. hirsutum*, but also for supporting the habitats of other endangered species and enhancing the overall biodiversity.

## Figures and Tables

**Table 1:** Vegetation and study site characteristics.

	TSD	JGR	JNC	SSR	CSR	Unit
Population	80	5	3	2	1	plant
Height of herbaceous layer	1.67 ± 0.47	1.33 ± 0.47	1 ± 0.27	1.27 ± 0.26	0.93 ± 0.19	m
Distance from woody plants	9.67 ± 0.47	2 ± 0	5.33 ± 0.47	10 ± 0	6 ± 0	m
Distance from water bodies	0.13 ± 0.09	0.13 ± 0.05	2.8 ± 0	0.17 ± 0.05	3 ± 0.57	m
Waterline width	2 ± 0	2 ± 0	0.7 ± 0	0.37 ± 0.05	15.33 ± 2.49	m
Rock exposure	6.7 ± 9.4	8.3 ± 8.5	0 ± 0	26.7 ± 12.5	6.7 ± 6.2	%
Bare land exposure	0 ± 0	1.7 ± 2.4	0 ± 0	8.3 ± 2.4	16.7 ± 6.2	%
Slope	28.3 ± 10.3	5 ± 0	0 ± 0	25 ± 4.1	13.3 ± 6.2	°
Soil depth	28.3 ± 2.4	21.7 ± 2.4	20 ± 0	10 ± 0	11.7 ± 2.4	cm

**Table 2:** The results of soil and water quality analysis at the study sites.

	Abbreviation	TSD	JGR	JNC	SSR	CSR	Unit
Soil pH	SpH	6.9 ± 0.03	6.78 ± 0.08	6.66 ± 0.06	6.57 ± 0.01	6.23 ± 0.27	
Soil humidity	SH	76.7 ± 2.7	67.8 ± 3.1	73.3 ± 9.8	76.9 ± 2.5	68.9 ± 22	%
Soil organic matter	SOM	6.66	4.3	1.95	3.16	2.44	%
Effective phosphoric acid	4	295.32	95.42	40.08	32.44	31.81	mg/kg
Cation exchange capacity	CEC	25.84 *	15.7	12.5	7.3	7.4	cmol^+^/kg
K	6	0.28 *	0.21	0.31	0.47	0.29	cmol^+^/kg
Ca	7	13.03 *	17.33	10.44	10.05	5.97	cmol^+^/kg
Mg	8	3.73 *	1.11	1.12	2.77	0.9	cmol^+^/kg
Na	9	0.05 *	0.29	0.07	0.26	0.12	cmol^+^/kg
EC	10	280 *	180	70	140	80	µS/cm
Sand	11	40.2 *	79.85	53.78	87.56	89.14	%
Silt	12	44.7 *	9.28	26.7	3.9	1.53	%
Clay	13	15.1 *	10.87	19.52	8.54	9.33	%
Texture	14	Loam *	Sandy loam	Sandy loam	Loamy sand	Loamy sand	-
Water DO	WDO	11.26 ± 0.38	10.05 ± 0.01	7.17 ± 0.04	9.22 ± 0.08	9.2 ± 0.04	mg/L
pH	WpH	7.43 ± 0.06	6.7 ± 0.07	6.85 ± 0.25	6.32 ± 0.24	6.97 ± 0.01	
Water Electrical conductivity	WEC	354 ± 1.4	347.9 ± 4	234.8 ± 18.1	314.1 ± 0.2	55 ± 0.9	µS/cm
Water hydraulic pressure	WPre	695.53 ± 0.05	701.53 ± 0.05	711.8 ± 0	744.87 ± 0.05	734.5 ± 0	mmHg

* The data for TSD are partially based on the findings of Cho et al. [14].

**Table 3:** Water quality characteristics of streams near the study site.

	Abb.	TSD	JGR	JNC	SSR	CSR	Unit
DO	WDO	11.26 ± 0.38	10.05 ± 0.01	7.17 ± 0.04	9.22 ± 0.08	9.2 ± 0.04	mg/L
pH	WpH	7.43 ± 0.06	6.7 ± 0.07	6.85 ± 0.25	6.32 ± 0.24	6.97 ± 0.01	
EC	WEC	354 ± 1.4	347.9 ± 4	234.8 ± 18.1	314.1 ± 0.2	55 ± 0.9	µS/cm
Water hydraulic pressure	WPre	695.4 ± 0.2	701.5 ± 0.1	711.7 ± 0.3	774.9 ± 0.1	734.5 ± 0.1	mmHg

**Table 4:** Factor analysis between *E. hirsutum* population and site characteristics.

	Factor 1	Factor 2
Population	0.8351 **	−0.4649 *
Slope	0.1890	−0.8946 **
Soil pH	0.8212 **	0.2351
Soil humidity	0.0134	−0.6106 *
Effective soil depth	0.9572 **	0.1161
Mean March temperature	−0.8827 **	−0.3784 *
Water vapor pressure	−0.9324 **	−0.2688
Effective phosphoric acid	0.9041 **	−0.3576 *

Definition of symbol: *, Factor loadings > 0.3; **, Factor loadings > 0.7.

## Data Availability

Data are unavailable due to privacy and ethical restrictions.
